# Maximizing flow rate in single paper layer, rapid flow microfluidic paper-based analytical devices

**DOI:** 10.1007/s10404-023-02679-8

**Published:** 2023-09-13

**Authors:** Iain Macleod Briongos, Zachary D. Call, Charles S. Henry, David L. Bark

**Affiliations:** 1 School of Biomedical Engineering, Colorado State University, Fort Collins, CO 80523 USA; 2 Department of Chemistry, Colorado State University, Fort Collins, CO 80523 USA; 3 Division of Hematology and Oncology, Department of Pediatrics, Washington University in St. Louis, St. Louis, MO 63108 USA; 4 Department of Biomedical Engineering, Washington University in St. Louis, St. Louis, USA

**Keywords:** Point-of-care, Paper microfluidics, Low volume, Flow rate

## Abstract

Small, single-layer microfluidic paper-based analytical devices (µPADs) offer potential for a range of point-of-care applications; however, they have been limited to low flow rates. Here, we investigate the role of laser cutting paper channels in maximizing flow rate in small profile devices with limited fluid volumes. We demonstrate that branching, laser-cut grooves can provide a 59.23–73.98% improvement in flow rate over a single cut, and a 435% increase over paper alone. These design considerations can be applied to more complex microfluidic devices with the aim of increasing the flow rate, and could be used in stand-alone channels for self-pumping.

**Supplementary Information:**

The online version contains supplementary material available at 10.1007/s10404-023-02679-8.

## Introduction

Microfluidic paper-based analytical devices (µPADs) offer many advantages for use in point-of-care assays. The low cost of the components and the ease of larger-scale production allow for extremely inexpensive devices (Martinez et al. 2010; Berry et al. 2016). Multiple physical effects can be combined to manipulate fluid flow without the need of an external pump. The wicking nature of paper and hydrophilic plastics can be leveraged to promote flow through capillary action; hydrophobic wax barriers can effectively direct flow; and surface tension from the formation of a droplet at the inlet can increase driving pressure for flow (Channon et al. 2018; Elizalde et al. 2015; Yang et al. 2017; Carrilho et al. 2009; Berthier and Beebe 2007; Hong and Kim 2015). Key to a μPAD is that it does not require electricity, making it an ideal tool in developing countries and emergency situations. This feature is of particular benefit when considering the development of point-of-care microfluidic systems in a diverse range of biological and chemical applications, including infectious disease diagnostics (Martinez et al. 2007; Hamedpour et al. 2018; Soum et al. 2019) and liquid quality testing (Charbaji et al. 2021; Guan and Sun 2020; Nilghaz et al. 2021) that are optimized for ease of usage, simplicity, and deliverability to the end user.

µPADs have been limited to low-flow applications until recently, where cut-out channels have been shown to increase the speed of fluid traversing through a µPAD (Renault et al. 2013; Channon et al. 2018; Ren et al. 2019). Other work creates partial (Giokas et al. 2014) and complete cut grooves within paper to increase and control flow rates up to 30 µL/min (Sotoudegan et al. 2019), which is otherwise not possible with traditional paper wicking. Controlling flow rate enables the user to regulate transport relative to reaction kinetics when considering chemical or biological reactions (Roder et al. 2004; Chatterjee et al. 2010). High flow rates can increase the speed of otherwise slow assays, which can be important in some point-of-care applications that require the transport of a liquid to multiple reagents or reaction sites. High speeds can help maintain polymers or cells in suspension (Campos Marín et al. 2018), as well as increase the rate of sample mixing (Tan and Neild 2012). Conversely, limiting the flow rate using hydrophobic materials such as wax or paraffin, or through transverse cuts in paper can allow for separation of components, multiple assays on a single device, and more (Noh and Phillips 2010; Giokas et al. 2014). Other microfluidic applications are reliant on control over wall shear rates or wall shear stress, e.g., when investigating cellular interactions that involve slip bonds or catch-slip bonds, and therefore high speed flow may be necessary to study specific cell-surface interactions (Pierres et al. 2008; Zhou et al. 2021; Marshall et al. 2003).

In fast flow µPADs, the relative contribution of each flow driver: channel geometry, dimensions, bifurcations, and confluences, have not been fully elucidated (Channon et al. 2019). Interdependent features could have net effects on the flow rate by counteracting or enhancing the effect of others. Methods that have been studied to obtain higher speeds involve using different types of paper with different porosities (Channon et al. 2019; Park et al. 2016); alterations to inlet size or volume of liquid provided or delivery method (Fu and Downs 2017; Ward et al. 2005); hydrophilicity to maximize capillary flow; size and presence of laser-cut grooves; and the overall channel dimensions, namely height, width, and length (Jang et al. 2020).

This work describes a stepwise method of configuring cut channels to maximize the flow rate in single paper layer devices. Single paper layers can be advantageous over multiple layers since the latter can introduce variability due to assembly challenges. Here, we compare the relative role of several parameters, while evaluating modifications to the channels to determine the most efficient ways of maximizing the flow rate within a self-driving paper-based pump, beyond increasing the number of channels, which was previously described (Sotoudegan et al. 2019). We obtain a flow rate that is 8 × greater for single paper layers. This can increase the speed of an assay in some applications; while in others, it may be necessary to maximize flow rates in a paper pump, e.g., to achieve high shear rates in a separate part of a channel to isolate specific shear-sensitive or flow-sensitive biological responses (Nesbitt et al. 2009). The resulting fast flow devices could be used independently, or combined with other µPAD design configurations for more complex applications.

## Methods

### Materials and equipment

Whatman grade 4 qualitative filter paper (Maidstone, UK) was used as the central component. Xerox Printer Wax (Norwalk, CT, USA) was used to print device borders. All devices were printed with a Xerox Colorqube 8870 or 8850 wax printer (Norwalk, CT, USA). Paper was cut using a 30-Watt Epilog Zing Laser Cutter and Engraver (Golden, CO, USA). 3 M Scotch laminator sheets (Saint Paul, MN, USA) were used to encase every device. All lamination was done using a Royal Sovereign IL-1346W (Rockleigh, NJ, USA). Inlet size was standardized using a Swingline hole punch (New York, NY, USA). Green colored dye (KTDORNS, Amazon) was perfused through the channels to test them.

### Device fabrication

**Fig. 1:**
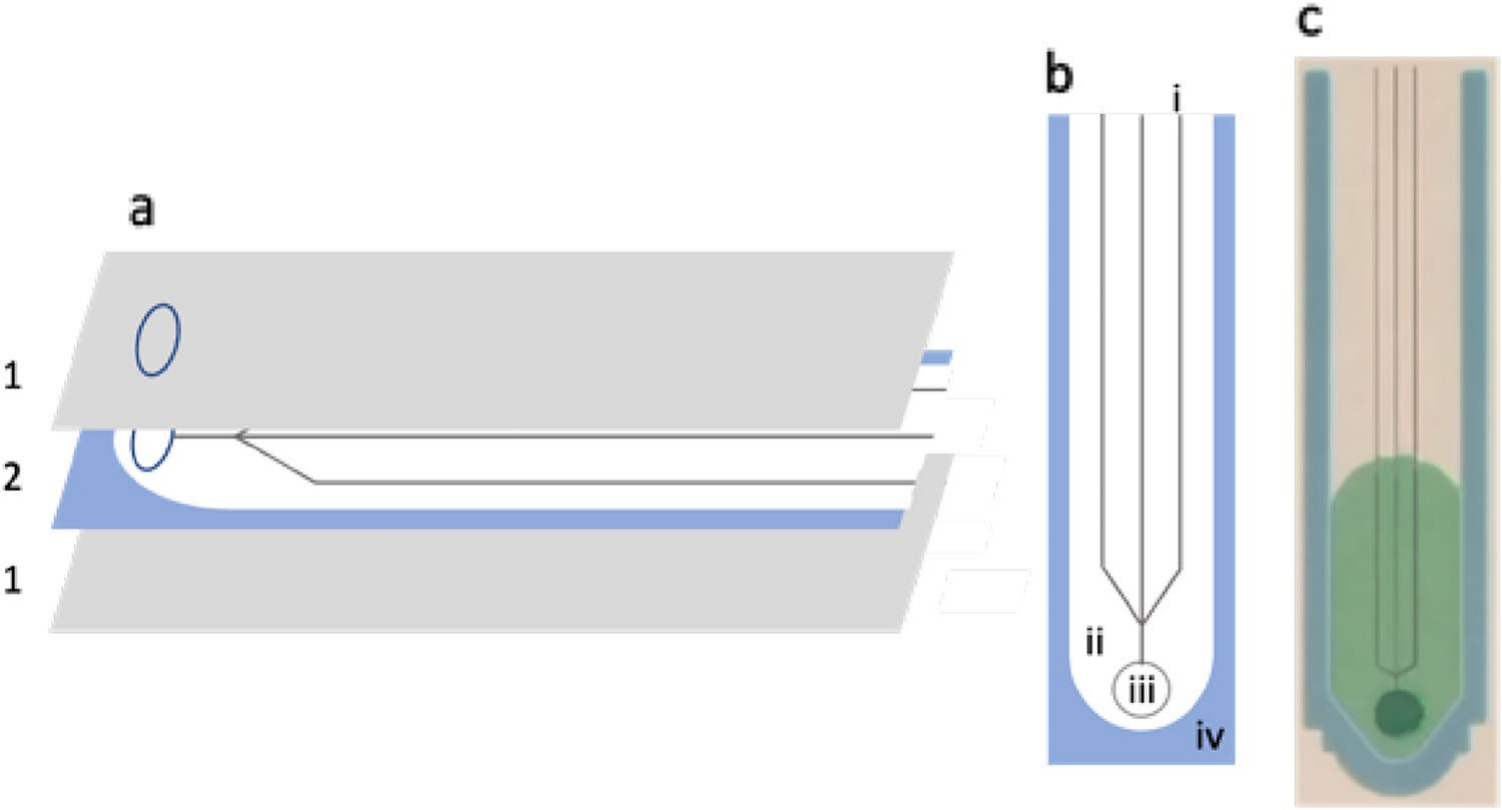
An overview of the channel construction. **a** Layer composition and orientation; 1, the laminate sheet; 2, Whatman 4 cellulose filter paper. **b** Paper layer schematic; i, laser-cut grooves; ii, the paper channel; iii, the inlet cut; iv, the wax perfused paper indicating the boundary of the channel. **c** An example of a physical channel showing dyed water flowing through the channel

An overview of the device is shown in Fig. 1. Devices were designed in CorelDRAW X4 (Corel, Ottawa, Canada). Whatman grade 4 qualitative paper was printed with colored wax, forming hydrophobic regions that surround hydrophilic channels of various widths. The printed wax was melted on an Isotemp hot plate (Fisher Scientific, Hampton, NH, USA) at 150 °C for 90 s, allowing it to perfuse through the paper, forming a hydrophobic barrier to contain and direct fluid flow. Device grooves, branches, and other features were designed in OnShape (Cambridge, MA, USA) which were then laser cut into the paper channels using a carbon dioxide laser engraver (Golden, CO, USA). As prior work has involved partially and completely cutting through the paper (Giokas et al. 2014; Sotoudegan et al. 2019), laser strength was decided based on experiments involving evaluation cuts (Fig. S1). Channels were put into 3 mm laminator sheets where all, but the bottom sheet layer were perforated with a ¼ inch hole punch. The sheets were laminated using the 3 mm setting. The far end from the inlet of each sheet was then cut-off to allow air to flow out and prevent the build-up of pressure that could otherwise limit flow. Post-fabrication, devices were excluded from the study if there were wax droplets in the channel area, if the laser cuts were not adequately centered in the channel, or if the inlet cut was not centered.

### Flow of liquid through channels

For easy visualization, 75 µl of green, food-dye-infused deionized water was perfused through the devices. To avoid dependency on pipetting speed, the pipetted water was first allowed to form a droplet at the pipette tip and then gently placed onto the inlet of channels.

### Image analysis and data processing

All images were captured using an Apple iPhone 12 (Cupertino, CA, USA) and converted for processing using FFmpeg (ffmpeg.org). Images were analyzed in FIJI ImageJ (Schindelin et al. 2012), where the total perfused area was found at specific time intervals. The leading edge of liquid is not uniform, and the grooves provide an open gap that holds a greater volume of liquid, relative to surrounding paper. Therefore, the grooves were analyzed by generating a kymograph in FIJI to determine liquid travel within the groove. The coordinates of the edge of the kymograph were used to generate lines in MATLAB (Mathworks, Natick, MA, USA) representative of the distance traveled. From these combined, flow rates were calculated using the following modification of the standard volumetric flow rate equation to account for paper porosity:

$${Q}=\frac{{\Delta A}}{{\Delta t}}{h}\left(1+\upzeta \left({P}-1\right)\right),\quad\mathrm{ where}\quad {\zeta }=\frac{{{A}}_{\mathrm{paper}}}{{A}},$$

where *Q* is the volumetric flow rate, *A* is the total area, *h* is the channel heigh, ζ is the area fraction of wicking paper relative to the total area bounded by wax, *P* is the porosity of the paper, and *t* is the time. The modification was used to control for different groove cut numbers and compositions. Graphs and statistical analyses were generated in Graphpad Prism (San Diego, CA, USA).

## Results and discussion

### Device channel width

The width of the wax-bounded paper channels can affect liquid wicking speeds (Hong and Kim 2015). Therefore, we investigated the specific effect on wax-bounded paper channels with a cut groove, with widths of 2.5–15 mm at 2.5 mm increments. Devices have a single, centered, straight-line laser-cut channel from the inlet, extending 80 mm, corresponding to the end of the paper.

**Fig. 2:**
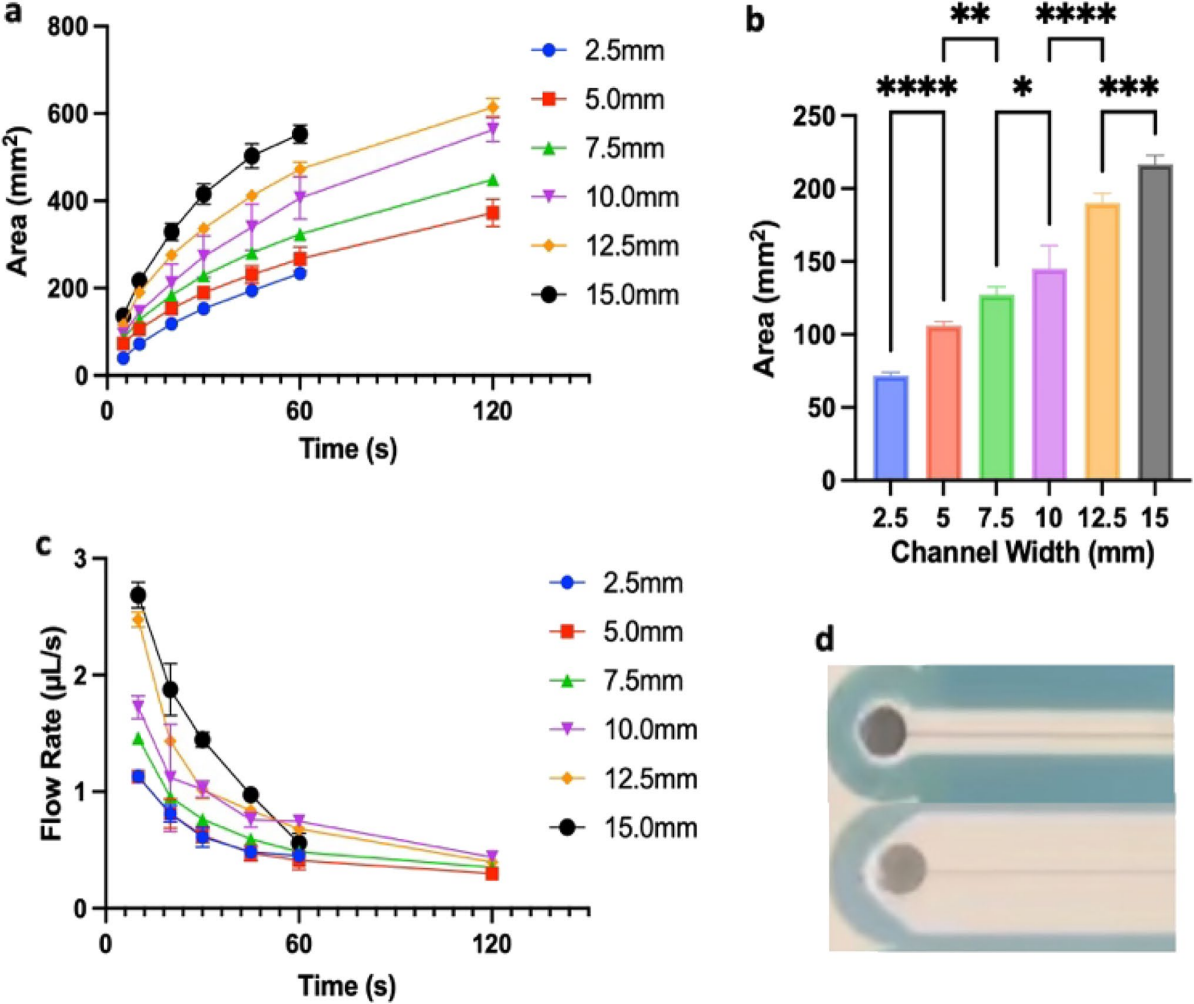
**a** Wetted area at a given time for different paper channel widths with a single laser cut. At indicated times, the area of travel was measured. **b** Area change over first 10 s for each channel width, representing fastest measured flow rates. **c** Flow rates at given times. **d** Example channels with widths of 5 mm and 15 mm. The absence of a data point at 120 s for the 2.5 mm channel is due to the dye traveling to the end of the channel prior to 120 s. Absence for the 15 mm channel is because all 75 µL of liquid wetted/filled the channel before 120 s. *p ≤ 0.05, **p ≤ 0.01, ***p ≤ 0.001, ****p < 0.0001. *n = *5 in all cases

As shown in Fig. 2, increasing paper channel width results in an increased average travel area relative to time, leading to an average flow rate of 1.12 and 2.70 µL/s, in the 2.5 mm and 15 mm channels, respectively, over the initial measured timeframe (5–10 s).

An increase in flow rate with channel width is in partial agreement with observations from (Hong and Kim 2015). This earlier work demonstrates that flow rates increase proportionally with wax-bounded paper strip width, albeit slightly reduced when compared to no wax boundary, i.e., a direct paper cut. It is noted that the effect of the wax boundary in Hong and Kim is reduced as the channel widens. This prior works also indicates that a 15 mm wide channel (without a groove) would have a flow rate that is more than 6 × a 2.5 mm channel. However, in the current work, a groove is cut through the centerline of the channel. This leads to an overall faster flow rate, but one that does not increase proportionally with channel width, Fig. 2, e.g., a 15 mm channel has a flow rate less than 3 × the 2.5 mm, as opposed to the predicted 6 × from Hong and Kim. We attribute this to the groove, which minimizes viscous resistance, while increasing the capillary pressure along the groove. As shown in supplementary materials (Fig. S1), the liquid fills the groove first early during flow, as wetting proceeds perpendicular to the groove. The liquid does not proceed with a uniform, flat leading edge like it does with no groove, as seen in Hong and Kim. Since the flow rate is most affected by the groove in the region local to the groove, it greatly enhances flow rates for thinner channel widths, with less of an effect on greater channel widths where boundaries are far from the centerline groove. Results demonstrate that increased channel width will increase flow rates (although not proportionally), but grooves may need to be distributed along the channel to maximize the flow rate, as has been done by others (Sotoudegan et al. 2019).

While liquid flows into both paper and the groove as soon as it is added to the inlet, it initially flows faster into the groove, compared to the rest of the paper, similar to (Sotoudegan et al. 2019). This effect is distance based, with liquid travel distance being proportional to the square root of time. At the same time liquid travels down the groove, liquid imbibition occurs in the paper, albeit more slowly, supplementary materials (Fig. S1). This appears to be contrary to what was found by Sotoudegan et al., where grooves filled completely before the majority of the paper was wetted. This difference could be due the difference in viscosity of blood and water or could depend on the presence of transparency layers in the current work.

### Groove width

**Fig. 3:**
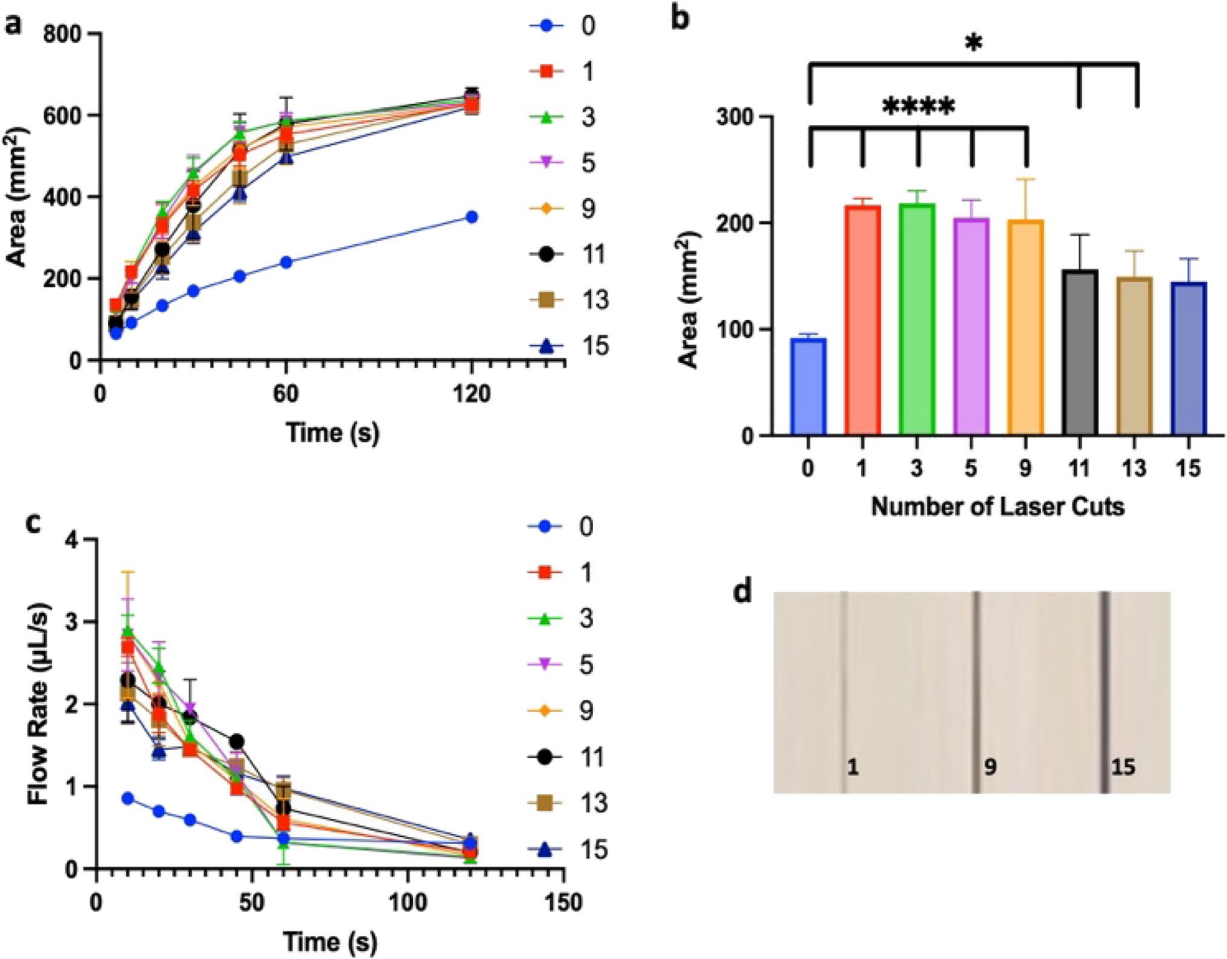
**a** Wetted area at a given time for different 15 mm wax-sealed paper channels with a range of laser cuts (1–15). At indicated times, the area was measured in FIJI. **b** Area change over the first 10 s for each channel width, representing fastest measured flow rates. **c** Flow rates at given times (**a**). **d** Example cut widths for 1, 9, and 15 laser passes, or 265.94 ± 30.83, 785.48 ± 27.92, and 1070.42 ± 26.90 µm, respectively. *p ≤ 0.05, ****p < 0.0001. *n = *5 for (**a**–**c**) and *n = *9 for **d**

To investigate the impact of groove width on flow rates, 15 mm channels were made with a central laser cut (groove) that was varied in width. For this purpose, 1–15 parallel, partially overlapping laser cuts, 0.05 mm apart were added. Laser passes were overlapped to minimize the amount of loose paper threads remaining after the cutting process and power was increased until the laser intensity led to a clean cut through the paper width, supplementary materials (Fig. S2). Measured groove widths are reported in supplementary materials (Fig. S3). Area to time curves exhibit similar responses at all measured widths, all of which are greater than a channel with no groove, Fig. 3. Flow rate also appears relatively independent of groove width for the widths considered here. Flow rates relative to time within the groove, itself, are also provided in supplementary materials (Fig. S4). These follow a similar trend to Fig. 3c with the volume flow rate through the groove accounting for less than 5% of the overall flow rate. However, flow rates in paper channels with grooves are overall greater than 2 × when compared to channels without grooves.

Marked improvements in flow rate are seen until 9 cuts, corresponding to a groove width approaching 800 µm, which is almost 4 × the thickness, but this trend does not continue for wider grooves. The trend of increasing flow rate with increasing groove size is similar to multilayer channels consisting of a groove within a double-sided adhesive layer sandwiched between two paper layers (Channon et al. 2018). Based on these results, we speculate that the groove helps to wet the paper along the length of liquid within the groove, leading to overall faster flow rates, but the groove, itself, provides little volume flow rate when compared to the combination of a groove with the paper channel.

### Branching grooves

**Fig. 4:**
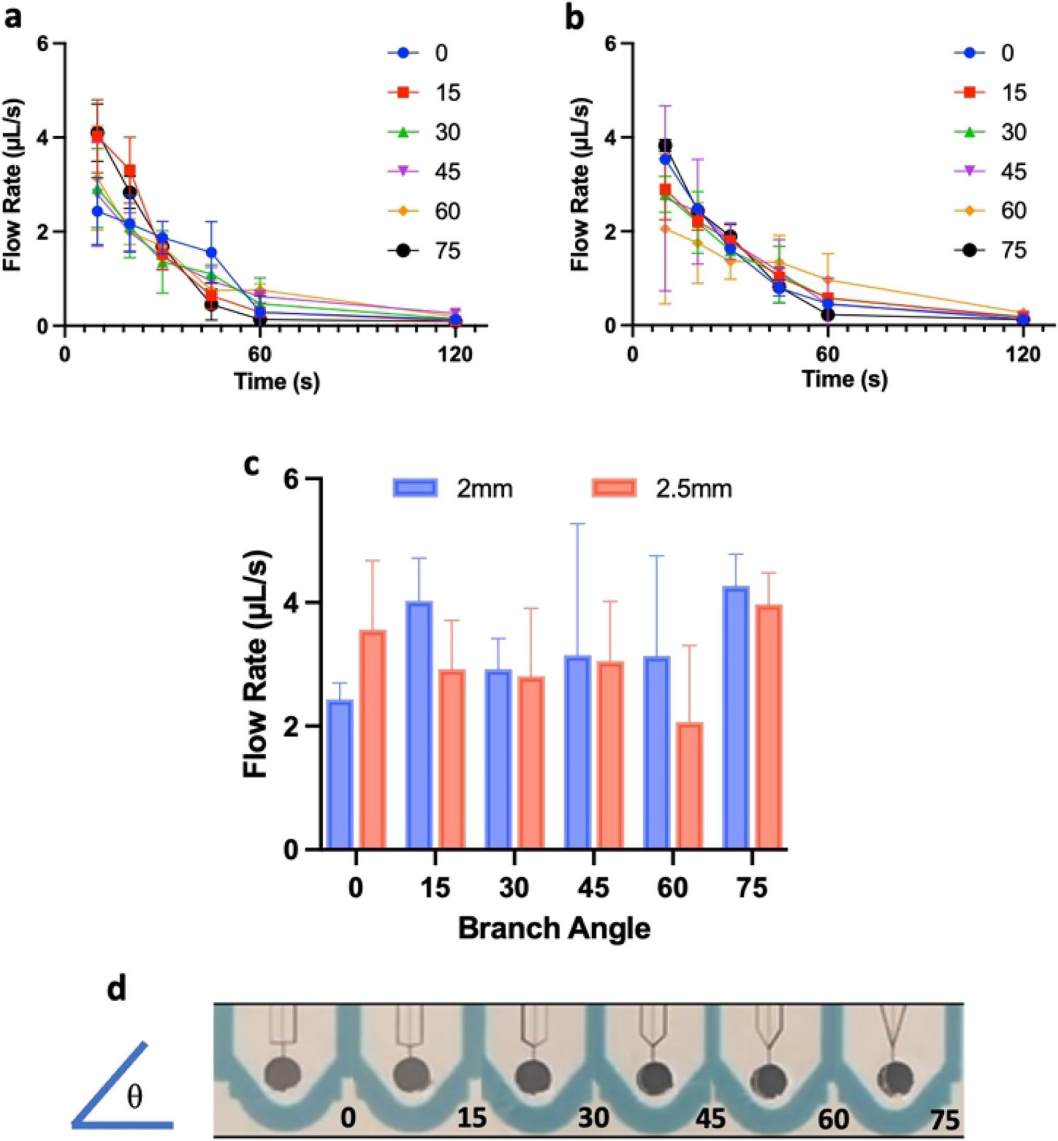
**a** 75 µL dyed water was flown through the channels and recorded for 2 mm branches. **b** The same test for branch spacing of 2.5 mm. **c** Comparison of peak flow rates (10 s) between 2 and 2.5 mm branch channels. **d** example series 2.5 mm branching cuts showing how they connect to the central groove going from 0 degrees (left) to 75 degrees (right) from horizontal. *n = *5 in all cases

Since liquid flows faster with the grooves compared to paper alone, we investigated the possibility that branching grooves could increase the speed of flow. We varied two factors: the angle at which branches join to the central groove and the spacing between branched grooves with results shown in Fig. 4. The ideal branch angle will cause minimal interference of the flow along the central groove and will also encourage flow along branching grooves. It is noted that surface tension at the air–liquid interface at the leading edge of the liquid would need to be overcome or the paper at the trifurcation would need to be wetted in order for liquid to move past the branching point. Two side-branching grooves were created in 15 mm channels, equally spaced to either side of the central groove with which they intersect at angles of 15–90 degrees, with 15-degree intervals. Additionally, the spacing between the branches was investigated, with flow rates from a branch spacing of 2 mm shown in Fig. 4a and a branch spacing of 2.5 mm shown in Fig. 4b. At certain spacing, there may be higher wicking potential, as fluid can wet the paper more uniformly across and between branches.

**Fig. 5:**
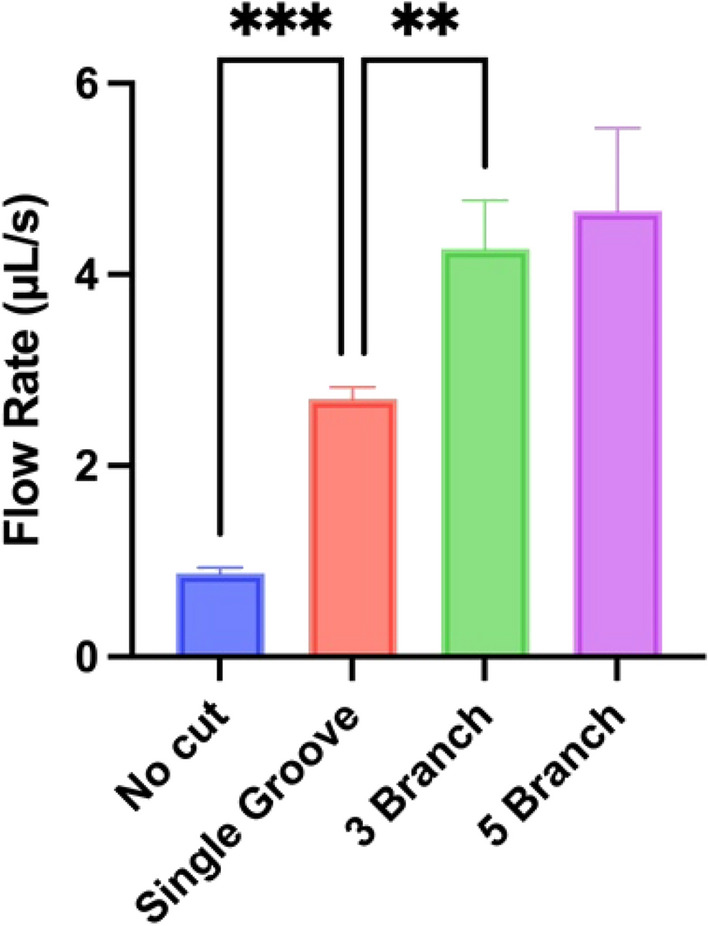
Comparison of best result flow rates from for 15 mm channels with no groove, compared with 1, 3, or 5 grooves. **p ≤ 0.01, ***p < 0.001

The addition of trifurcating grooves led to an improvement in the area covered per second, translating to the average peak flow rate increasing from 2.70 µL/s in the 15 mm, single central groove, to 3.97 µL/s and 4.27 µL/s with the addition of 75-degree, 2.5 mm- and 2.0 mm-spaced grooved branches, respectively. Note that not all branches offered an improvement, with some, such as the 0-degree branch causing a decrease in the flow rate to 2.43 µL/s. The additional groove branches could interfere with the uniformity of flow, leading to a greater variability than with a single central groove only (Fig. S1). The two 75-degree branches led to fastest flow rates with least variability. Note that while the area change between a single cut and both 75-degree cuts was not striking, the change in flow rate was found to be statistically significant (see Fig. 5). The best configuration of branched cuts was then tested in 15 mm channels along with an additional two 2 mm-spaced grooves (bringing the total up to 5 branches).

While the addition of two further grooved branches did increase the average peak flow rate to 4.66µL/s, it comes with the caveat that the flow was significantly more variable. As the number of grooves increases, the paths within which liquid can flow increases, leading to lower channel resistance and faster flow. However, the surface tension at the air–liquid interface needs to be overcome. Non-uniformities in the paper and laser cutting could influence the timing of the split into multiple branches. These factors could lead to greater variability in flow profiles (Fig. S1) and to flow rate values calculated for Fig. 5. Over the single-grooved 15 mm channels, the 3-branch and 5-branch channels provided a 59.23% and a 73.98% increase in the flow rate, respectively, primarily due to a flatter leading edge for the liquid when compared to a single groove. It is noted that although additional grooves increase the flow rate, Fig. 5, the velocity of single grooves within the multi-groove design does not increase markedly compared to a single groove. Instead, additional grooves appear to enhance the perfusion of liquid through the surrounding paper along the width of the channel.

### Flow effects

To develop further insight into how the grooves impact flow through single-layer µPADs, theoretical analyses are used. Most theory has stemmed from the Lucas-Washburn equation, commonly used to describe sample flow in µPADs,

$${l}\left({t}\right)=\sqrt{\frac{\upgamma \cdot {r} \cdot {t} \cdot {cos}\left(\uptheta \right)}{2\upmu }},$$

where *l* is the distance traveled down the channel length at time *t*, *γ* is the interfacial tension, *r* is the effective capillary radius, *θ* is the contact angle of the fluid to the surface, and *µ* is the viscosity. Although it was developed for flow through a capillary, it has been accurate in predicting flow through µPADs. The equation can be extended to by considering capillary flow in bundles of parallel tubes, which approximately represents interwoven paper fibers. Iterations on this equation are also common to account for complex designs that go beyond single paper layer µPADs. Generally, a constant in front of the equation can be adjusted to accommodate the tortuosity of flow through paper and to accommodate the additional complexities.

**Fig. 6:**
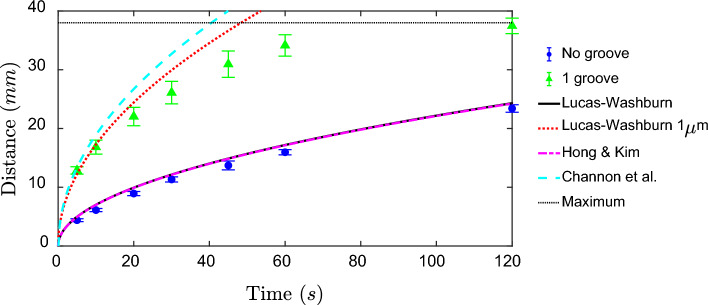
Representative average distance of the leading front of dyed water in 15 mm wide microfluidic devices with listed parameters. Note that the maximum distance flow can travel is denoted by the black dotted line

Flow in the presence of a wax border has been shown to be slower than when compared to paper alone and when compared to the Lucas–Washburn equation (Hong and Kim 2015) due to a reduced capillary effect at the boundary caused by the > 90° contact angle between water and the hydrophobic wax. However, for wide channels of 15 mm, the wax boundary is predicted to minimally affect flow, Figs. 2, 6. In this case, the Hong and Kim equation:

$$\underset{{w}\to \infty }{{lim}}{l}={k}\sqrt{\left(1+\frac{{\beta d}}{{w}{\upphi }^\frac{1}{3}}\frac{{cos}{\uptheta }_{{b}}}{{cos\theta }}\right)\frac{\upgamma }{\upmu }{t}}={k}\sqrt{\frac{\upgamma }{\upmu }{t}}$$

simplifies to the Lucas–Washburn equation for wide channels, where *k* is a proportional constant, $\upphi$ is the porosity, *w* is the channel width, $\upbeta$ accounts for the length of contact lines with a wax boundary, and ${{\uptheta }_{\mathrm{b}}}$ is the contact angle with the wax boundary. Both Lucas–Washburn and the Hong and Kim equations matched well with experiments lacking a groove, Fig. 6.

With the groove, distance squared is proportional to time, which suggests a Lucas–Washburn-like behavior. If we use a pore radius of 1 µm, results match closely, but exceed values for a single groove. A previous relationship was developed for flow through a multi-layered cut-out groove (Channon et al. 2019):

$${t}{\left(\frac{{dl}}{{dt}}\right)}^{2}+\frac{3{{t}}_{{h}}^{2}}{{{h}}^{2}}{l}\frac{{dl}}{{dt}}=\frac{\upgamma {{t}}_{\mathrm{h}}^{2}{{cos}}^{2}\uptheta }{{\mu h}},$$

where ${{t}}_{\mathrm{h}}$ is the thickness of the paper and *h* is the half channel height for multi-layered devices. In the current work, *h* is taken as half of the groove width and the equation was numerically solved. This equation also over-predicts flow through the grooved single paper layer. Therefore, the groove may not be as effective as a multi-layered device. Compared to flow through the multi-layered channels, it is not expected that the leading edge of flow in the groove will have a parabolic profile. Instead the meniscus is likely localized to area near the paper boundary. Therefore, *h* may be overestimated by assuming it is half of the groove width. Furthermore, laminating the device has been previously shown to reduce flow by more than half compared to open channels (Channon et al. 2018). Overall, the groove in single-layer devices exhibits similar flow behavior as a groove in a multi-layered rapid flow device.

## Conclusions

Herein, we detail a procedural method for the increasing the flow rate in small profile, single-layer, microfluidic devices. Increasing the area of available paper for wicking leads to a marked improvement in flow rate. Through the addition of a single laser-cut groove down a channel from the inlet, volumetric flow rates can be improved by 435% when compared to an equivalent channel with no groove (i.e., paper only). Flow rates reach a peak of 4.66 µl/s, which to our knowledge is the highest in a single-layer device (Table S1). Further addition of grooved branches from the central branch leads to an increase in the flow rate of 59.23% for 3 branches and 73.98% for 5 branches.

It is possible that flow rate could be further improved by controlling cellulose fiber composition more completely than with commercially available papers (Böhm et al. 2014), as well as by modifying channel height (Channon et al. 2018), width or length (Hong and Kim 2015; Songok and Toivakka 2016).

The devices described here could be used as a wicking pad for other µPAD configurations that require high flow rates such as cellular testing and point-of-care applications, slip and catch–slip bond investigation (Marshall et al. 2003; Zhou et al. 2021; Christophis et al. 2011), increased mixing rates (Tan and Neild 2012; Ward and Fan 2015) and more.

### Supplementary Information

Below is the link to the electronic supplementary material.

Supplementary file1 (DOCX 4337 kb)

## Data Availability

The datasets generated during and/or analyzed during the current study are available from the corresponding author on reasonable request.
