# Supplementary material for: Maximizing flow rate in single paper layer, rapid flow microfluidic paper-based analytical devices
Source: Microfluid Nanofluidics. 2023 Sep 13;27(10):70. doi: 10.1007/s10404-023-02679-8 (PMC10499984; doi:10.1007/s10404-023-02679-8)
Supplement: Supplementary file 1 — Supplementary file1 (DOCX 4337 kb) [file 10404_2023_2679_MOESM1_ESM.docx]

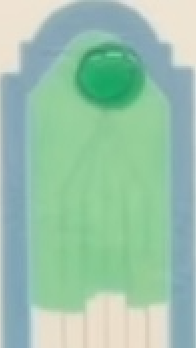

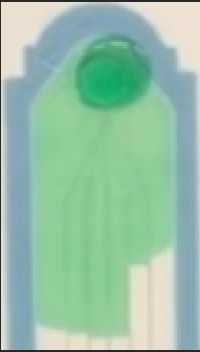

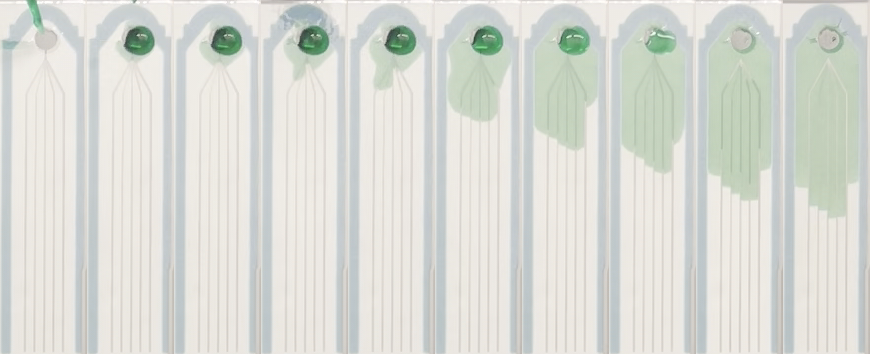

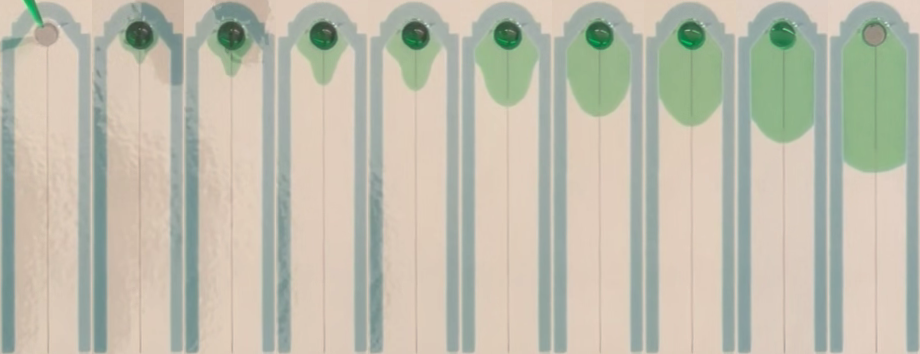

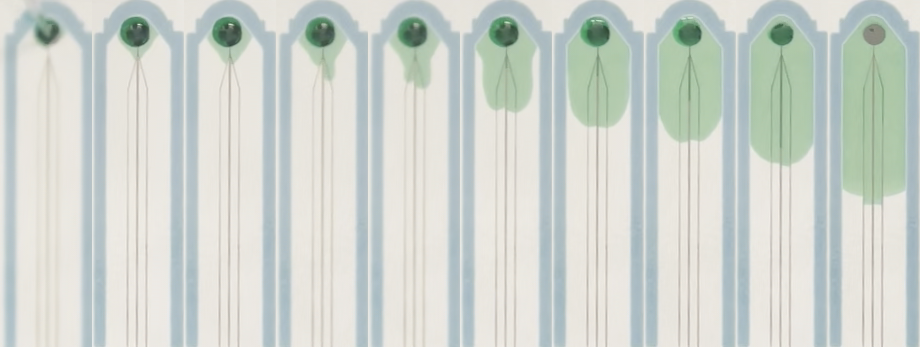

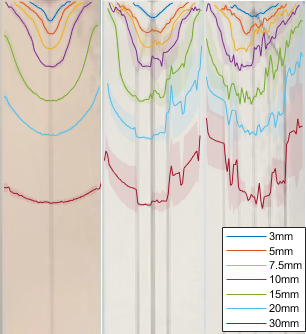


0s 1s 2s 3s 4s 10s 15s 20s 30s 60s

S1. Timelapse displaying how liquid flows in channels. Left- 15mm channel with single, 3-branch and 5-branch grooves shown at 0, 1, 2, 3, 4, 10, 15, 20, 30 and 60s. Top right – Liquid flow profile at indicated distances from the inlet with standard deviation cloud (n=4). 3mm marks the branch point. Bottom right – Variability of channels increases with additional branches illustrated by two 5-branch channels shown at same timepoint.

S2: To compare ideal laser cut strength, identical channels were tested where groove was cut at different laser intensities. At 9% laser intensity, the paper was fully cut through.

S3: Width of grooves. Overlapping grooves were laser cut into channels and measured under microscope (n=5 with 3 measurements at different points per n).


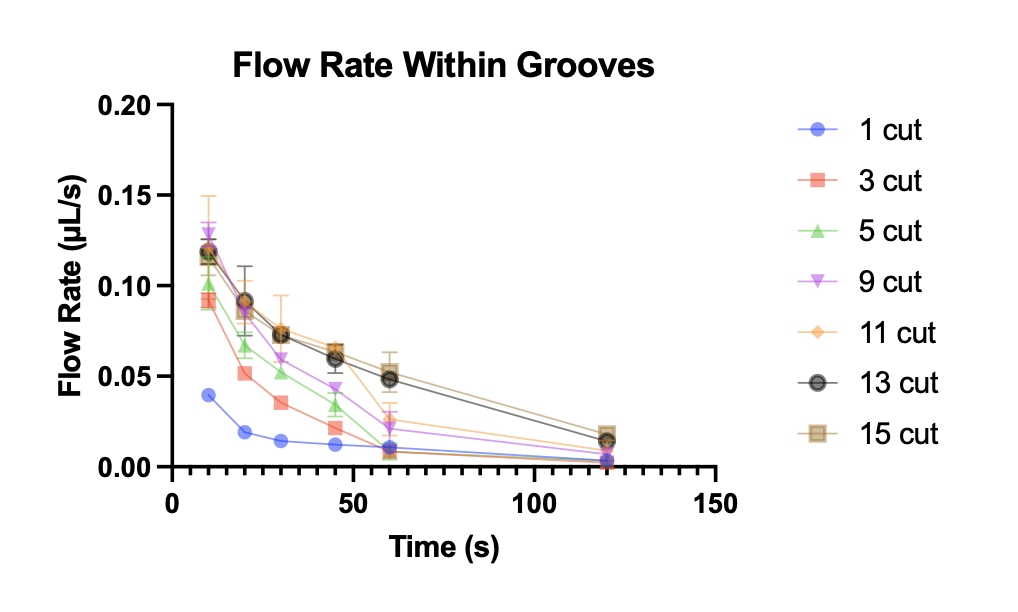


S4. Flow rate within grooves of widths shown in S2. N=5

| **Method** | **Peak Flow Rate (µl/s)** | **Reference** |
| --- | --- | --- |
| Laser cut grooves | 4.66 | This paper |
| Pressurised (4mbar) inlet into channels in paper | 3.50 | (Renault et al. 2013) |
| Laser cut with feeding channel | 2.00 | (Sotoudegan et al. 2019)* |
| Partially cut channels | 1.67 | (Giokas et al. 2014) |
| Paper pump | 0.49 | (Evans et al. 2014) |
| Capillary pumping | 0.40 | (Guo et al. 2017) |
| PET covered channels | 0.075 | (Jahanshahi-Anbuhi et al. 2012) |

Table S1. Estimated peak flow rate comparison with range of similar devices. All using water, except *, which has been converted from blood.

**References**

Evans E, Gabriel EFM, Coltro WKT, Garcia CD (2014) Rational selection of substrates to improve color intensity and uniformity on microfluidic paper-based analytical devices. Analyst 139:2127–2132. https://doi.org/10.1039/C4AN00230J

Giokas DL, Tsogas GZ, Vlessidis AG (2014) Programming Fluid Transport in Paper-Based Microfluidic Devices Using Razor-Crafted Open Channels. Anal Chem 86:6202–6207. https://doi.org/10.1021/ac501273v

Guo W, Hansson J, van der Wijngaart W (2017) Capillary pumping with a constant flow rate independent of the liquid sample viscosity and surface energy. In: 2017 IEEE 30th International Conference on Micro Electro Mechanical Systems (MEMS). IEEE, pp 339–341

Jahanshahi-Anbuhi S, Chavan P, Sicard C, et al (2012) Creating fast flow channels in paper fluidic devices to control timing of sequential reactions. Lab Chip 12:5079. https://doi.org/10.1039/c2lc41005b

Renault C, Li X, Fosdick SE, Crooks RM (2013) Hollow-Channel Paper Analytical Devices. Anal Chem 85:7976–7979. https://doi.org/10.1021/ac401786h

Sotoudegan MS, Mohd O, Ligler FS, Walker GM (2019) Paper-based passive pumps to generate controllable whole blood flow through microfluidic devices. Lab Chip 19:3787–3795. https://doi.org/10.1039/C9LC00822E
